# Overview of Renal Replacement Therapy Use in a General Intensive Care Unit

**DOI:** 10.3390/ijerph19042453

**Published:** 2022-02-21

**Authors:** Mirela Tiglis, Ileana Peride, Iulia Alexandra Florea, Andrei Niculae, Lucian Cristian Petcu, Tiberiu Paul Neagu, Ionel Alexandru Checherita, Ioana Marina Grintescu

**Affiliations:** 1 Department of Anesthesia and Intensive Care, Emergency Clinical Hospital of Bucharest, 014461 Bucharest, Romania; mirelatiglis@gmail.com (M.T.); iulia.alexandra.florea@gmail.com (I.A.F.); ioana.grintescu@umfcd.ro (I.M.G.); 2 Clinical Department No. 14, “Carol Davila” University of Medicine and Pharmacy, 020021 Bucharest, Romania; 3 Clinical Department No. 3, “Carol Davila” University of Medicine and Pharmacy, 020021 Bucharest, Romania; ileana_peride@yahoo.com (I.P.); niculaeandrei@yahoo.com (A.N.); al.checherita@gmail.com (I.A.C.); 4 Department of Biophysics and Biostatistics, Faculty of Dentistry, “Ovidius” University, 900684 Constanta, Romania; crilucpetcu@gmail.com; 5 Clinical Department No. 11, “Carol Davila” University of Medicine and Pharmacy, 020021 Bucharest, Romania

**Keywords:** critical care, renal replacement therapy, ICU scores, length of ICU stay, mortality

## Abstract

Objectives. Population-based studies regarding renal replacement therapy (RRT) used in critical care populations are useful to understand the trend and impact of medical care interventions. We describe the use of RRT and associated outcomes (mortality and length of intensive care stay) in a level 1 hospital. Design. A retrospective descriptive observational study. Patients. Critically ill patients admitted to the ICU from 1 January to 31 December 2018. Interventions. Age, gender, ward of admission, primary organ dysfunction at admission, length of hospital stay (LOS), mechanical ventilation, APACHE, SOFA and ISS scores, the use of vasopressors, transfusion, RRT and the number of RRT sessions were extracted. Results. 1703 critically ill patients were divided into two groups: the RRT-group (238 patients) and the non-RRT group (1465 patients). The mean age was 63.58 ± 17.52 (SD) in the final ICU studied patients (64.72 ± 16.64 SD in the RRT-group), 60.5% being male. Patients admitted from general surgery ward needing RRT were 41.4%. The specific scores, the use of vasopressors, transfusions and mortality were higher in the RRT-group. The ICU LOS was superior in the RRT-group, regardless of the primary organ dysfunction. Conclusions. RRT was practiced in 13.9% of patients (especially after age of 61), with mortality being the outcome for 66.8% of the RRT-group patients. All analyzed data were higher in the RRT group, especially for multiple trauma and surgical patients, or patients presenting cardiac or renal dysfunctions at admission. We found significant increased ISS scores in the RRT-group, a significant association between the need of vasopressors or transfusion requirement and RRT use, and an association in the number of RRT sessions and LOS (*p* < 0.001).

## 1. Introduction

From all accounts, acute kidney injury (AKI) is a common complication of critically ill patients, being associated with grater morbidity and mortality rates, both at short and long terms, and this remains a constant health problem [1,2]. Approximately half of all intensive care unit (ICU) patients will go through at least one episode of AKI during hospitalization [3]. In critically ill patients, renal replacement therapy (RRT) is used to provide support for AKI or multiple organ dysfunction syndrome (MODS) [4,5]. Various reports have shown broad variability in estimating the use of RRT in ICU patients, from 5–10% to 38% or even 59.2%, with a trend of 10% increase per year in light of the continuous change in the critically ill patient profile [6,7,8,9,10].

The main indications of starting RRT in patients diagnosed with AKI or acute-on-chronic kidney disease are: signs of uremia (i.e., pericarditis, pleuritis, encephalopathy, etc.), hypervolemia nonresponsive to conservative therapy, severe hyperkalemia and/or metabolic acidosis, and drug intoxications [11].

In addition, reported main risk factors for the use of RRT in ICU patients are older patients, male gender, mechanical ventilation, important underlying cardiovascular disease, cardiac surgery, and hepatic failure [8,12]. The RRT use among ICU patients leads to increased length of stay (LOS), varying from 21 to 102 days [13,14], and to mortality rates up to 100% [15,16].

Although there are limited data about the costs related to RRT use in critically ill patients [17], in association with important mortality and morbidity, identifying patients with higher odds of benefitting after such intervention may be of great value for the improvement of medical decisions. Therefore, population-based studies regarding RRT use in the general critical care population are useful in understanding the trend and impact of medical care interventions.

The primary objectives of the study were to analyze the main characteristics of all patients’ admitted to an ICU from a level 1 emergency hospital during one year. The secondary objectives were to study mortality trends among those receiving RRT, the association between the medical wards from which patients were transferred to ICU and the need for RRT, and to identify the differences in length of ICU stay among patients with RRT, depending on the primary organ dysfunction at ICU admission.

## 2. Materials and Methods

### 2.1. Patient Selection and Data Collection

In this retrospective observational study, we have analyzed medical records of all critically ill patients admitted to the ICU of the Emergency Clinical Hospital of Bucharest from 1 January to 31 December 2018. Patients with CKD (chronic kidney disease) in a hemodialysis program continued their usual schedule at the Dialysis Unit and were not included in this analysis. Exclusion criteria were represented only by missing medical records from the reviewed ICU-database.

The variables defined for the statistical analysis were: age, gender, the ICU admission from various medical specialties (wards), the initial organ failure at admission, length of ICU stay (LOS), days of mechanical ventilation, APACHE II (Acute Physiology and Chronic Health Evaluation), SOFA (Sequential Organ Failure Assessment) and ISS (Injury Severity Score) scores at ICU admission, the use of vasopressor support or transfusion, the use of RRT and the number of RRT sessions. Overall and short-term mortality (in the first 48 h after admission) were also studied.

The study received the approval (no. 308/2022) of the Ethical Committee of the Emergency Clinical Hospital of Bucharest. Due to the non-interventional retrospective type of study and the use of anonymous data, the ethic committee waived the need for patient consent (ICU-database analysis).

### 2.2. Statistical Analysis

The statistical analysis was performed using IBM SPSS statistics software version 23. Data are presented as mean ± standard deviation (SD) for continuous variables in case of symmetric distributions, median and IQR (Interquartile range) for continuous variables in case of skewed distributions, or as percentages for categorical variables. The normality of the continuous data was estimated with Kolmogorov-Smirnov Tests of Normality. For hypotheses testing: independent samples *t*-Test, independent samples Mann Whitney U test, independent samples Median test, chi-squared test of association, and chi-squared test for the comparison of two proportions were used depending on the type of analyzed variables. Cox proportional-hazards regression, using the forward selection method, and Kaplan-Meier survival analysis was performed using MedCalc statistics software version 14.8.1. The probability of a Type I error (the significance level α) was set at 0.05. If the test statistic for every conducted test was in the critical region, and the *p*-value was less than or equal to the significance level, we decided to reject the null hypothesis in favor of the alternative hypothesis.

## 3. Results

**Figure 1:**
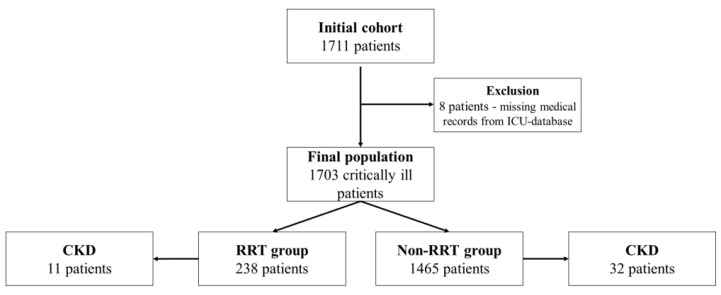
Flowchart. RRT—renal replacement therapy, CKD—chronic kidney disease.

Of the 1711 consecutive critically ill patients initially reviewed, eight patients were excluded from the analysis due to incomplete medical records in the ICU-database, leaving 1703 patients for the final analysis. Afterwards, patients were divided into two groups: the RRT group (patients with RRT during ICU stay)—238 patients (13.9%), the Non-RRT group (patients without RRT)—1465 patients (86.1%). In ICU, various types of continuous RRT (CRRT) were used. Regarding the presence of chronic kidney disease (CKD) at ICU admission, in the RRT group were 11 patients (4.6%), and in the non-RRT group there were 32 patients (2.2%) (Figure 1). None of the patients previously diagnosed with CKD and considered for this article were included in a chronic dialysis program.

### 3.1. Patient Demographics, Characteristics and the Use of RRT

The mean age of the Non-RRT group was 63.40 ± 16.65 SD, and it was 64.73 ± 16.65 SD for the RRT group, showing no significant statistical difference (*p* = 0.279). In Table 1 are presented the age intervals for both groups. The risk of needing RRT during an ICU stay is higher after 51 years of age. In the age intervals of 51–60, 61–70 and 71–80 we have observed a greater proportion of RRT use but we have not found statistically significant differences in the analyzed age groups (*p* = 0.324). Out of 1030 male patients (60.5%) of our studied group, only 149 (8.7%) were included in the RRT group. At the same time, out of 673 female patients (39.5%), merely 89 (5.2%) were included in the RRT-group. We also found that there is no association between the RRT use and patients’ gender (*p* = 0.470).

We state that there is an association between the RRT use and the presence of CKD, as we expected (*p* = 0.026). The risk for a patient with CKD to need RRT during ICU stay is 2.17 higher than the others (95% CI 1.078–4.366).

In Table 2, we present the main characteristics of the final included patients, regarding the ICU admission from various medical specialties (wards), the primary organ dysfunction at ICU admission, specific ICU scores at admission, mechanical ventilation use, vasopressor support and blood transfusion between both groups. Regarding the primary organ dysfunction, it was considered according to initial ICU diagnosis as being the main cause of ICU admission.

The analysed variables: ICU specific scores (APACHE II, ISS, SOFA), ICU stay, and days of mechanical ventilation are non-normally distributed (*p* < 0.05), therefore we have used non-parametric tests to assess if the distribution of scores for each variable are the same or not across our groups. Except for the days of mechanical ventilation (*p* = 0.315), all the other analysed variables showed significant differences between the distributions of scores and the median values across our groups (*p* ≤ 0.001).

We observed that only those proportions for which the significance level is <0.05 are significantly different, as with the proportion of patients coming from the General Surgery ward (*p* < 0.001) and patients coming from the Neurosurgery ward (*p* < 0.001), who were associated with risk of RRT use.

Similarly, we observed that only those proportions of patients presenting with renal dysfunction (*p* = 0.0031), gastrointestinal dysfunction (*p* = 0.0108), neurologic (*p* < 0.001), in postoperative status (*p* = 0.0123), or oncological status (*p* = 0.0110) as main dysfunction at ICU admission were significantly associated with the risk of needing RRT during the ICU stay.

Furthermore, transfusion requirements and the use of vasopressor therapy were correlated with the need of RRT in the studied population, with *p* < 0.0001 in both cases.

As expected, there was a statistically significant association between ICU stay and the number of RRT sessions, with a *p* < 0.001 (95% CI 0.26–0.48).

### 3.2. Mortality

After data analysis, 38.6% (*n* = 657) of the studied patients died during their ICU stay. In the RRT group, death was the outcome for 9.3% of patients (*n* = 159), while in the non-RRT group death was the outcome for 29.2% (*n* = 498) of the patients. For patients with CKD, 54.5% patients that required RRT and 42.4% patients from the non-RRT group died during ICU hospitalization. Table 3 presents the percentage of patients with fatal outcomes when also taking into account the primary organ dysfunction at ICU admission. Mortality within 48 h after admission was encountered in 15% of the cases in the RRT group, and 9.6% of the cases in the non-RRT group, respectively.

Additionally, we analyzed the association between mortality as the final outcome depending on the main organ dysfunction at ICU admission. Excepting cardiac dysfunction, all the others were positively associated with the risk of mortality during ICU stay (Table 3).

In order to analyze the variables that influence the risk of mortality in our patients, the Cox proportional-hazards regression model was applied in which the following variables were entered: age, gender, primary organ dysfunction, and RRT. Considering that APACHE II and SOFA scores were recorded for 1623 patients, and that there were only ISS scores for 161 patients out of the total of 1703 subjects, these variables could not be introduced in Cox proportional-hazards regression analysis, because when applying the procedure, the program eliminates records that have missing values. According to the model, age, male gender, the presence of cardiac, gastrointestinal, neurologic and renal dysfunctions, multiple trauma patients and the use of RRT influenced the mortality (overall model fit: Chi-squared = 172.053, df = 8, *p* < 0.0001). Table 4 presents the relative instantaneous risks (Exp(b)) of mortality occurrence for each of the analyzed variables. It should be noted that Exp(b) is going to be modified for each 1-unit change in the continuous variable only for continuous variables (age). Therefore, with ageing, the relative instantaneous risk of mortality will increase.

Regarding the Kaplan-Meier survival curves for the studied patients, the median value for the survival time was 12 days for the RRT group (95% CI 9.00–14.00) and 20 days for the non-RRT group (95% CI 17.00–26.00), these being significantly different (*p* < 0.001). Therefore, we can state that the RRT use significantly influences survival time, and patients requiring RRT during an ICU stay have a risk a of fatal outcome 1.62 times higher than for patients belonging to the non-RRT group.

**Figure 2:**
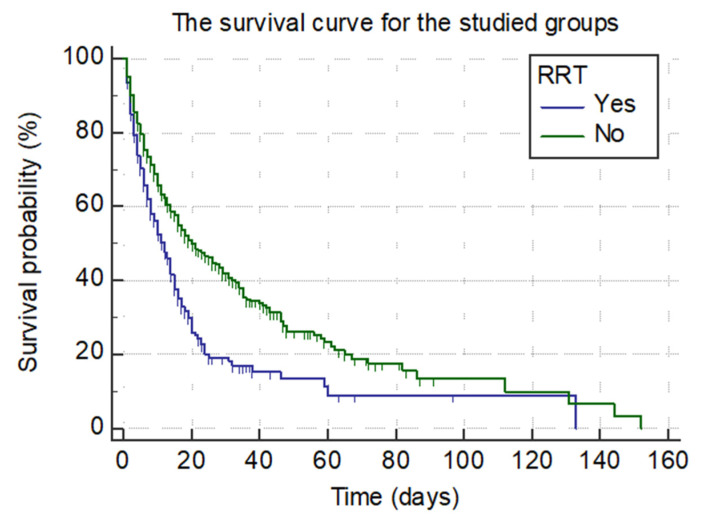
Survival curve for the RRT group and Non-RRT group (*n* = 1703).

The survival curves for both groups are presented in Figure 2.

In addition, we presented the survival proportion in various moments during the ICU stay (Table 5).

Therefore, the survival proportion decreases significantly after 12 days of ICU stay, both in the RRT and non-RRT groups.

### 3.3. Length of ICU Stay

In the RRT group, the mean length of ICU stay was 11.97 ± 14.76 SD days, and it was 9.77 ± 13.83 SD in the non-RRT group. We also analyzed the mean LOS stay according to primary organ dysfunction at admission. The results are presented in Table 6. Excepting patients with neurologic dysfunction upon ICU admission, the LOSs were lower in the non-RRT group.

After statistical analysis, we observed that there is a significant statistical difference between lengths of ICU stay and patients presenting with renal (*p* = 0.019) and gastro-intestinal dysfunction (*p* = 0.006), patients with multiple trauma (*p* = 0.041) or in post-operative status (*p* < 0.001) at ICU admission.

## 4. Discussion

Epidemiological studies regarding the use of RRT in critically ill patients are scarce, and usually focused on population characterization according to AKI presence and stage, main diagnosis/organ dysfunction, LOS or mortality depending on the AKI stage. Apart from these, we find it important to know the rate of RRT use among critically ill patients, not only in relation to the main (primary) organ dysfunction at ICU admission, but the leading medical specialties (wards) from which patients ends up in ICU, to be able to predict future trends. The use of vasopressor support, and blood products transfusion in patients receiving RRT, compared to those without this intervention, represent essential aspects of critically ill patient management.

The overall RRT need during ICU stay was 13.9% in our study. Others reported an incidence of 8.3%, Paccinni et al. [18], 8.8%, Fujii et al. [19], 12%, Harris et al. [20], and 20.4% was found by Oweis et al. [21]. The differences most likely result from the profile of each intensive therapy, the presented one being a general ICU unit.

A study published by Truche et al. included 5242 ICU patients and showed that the median age was 70.2 years [58.0–78.9]. Most patients had a medical condition (77.15%), shock (40.98%) and respiratory failure (22.57%) as the main dysfunctions upon admission [22]. In contrast, the median age in our study was lower, with a larger interquartile range, possibly because it is about a level 1 emergency hospital, with high accessibility. Furthermore, the primary cause for ICU admission was postoperative status (general surgery) (41.1%), followed by a medical condition (internal medicine) (24.3%); this trend was also encountered in the non-RRT group, along with orthopedic surgery (8.8%).

When it comes to the main characteristics of the ICU patients, as previous studies showed, there is an increased risk of using RRT in male patients, and the high ICU values of specific scores (SOFA, APACHE) are also associated with RRT use during ICU stay, elements also identified in this study [23,24].

Regarding mechanical ventilation use in patients receiving RRT, our finding is similar to other reported data, finding that it is frequently required in this category of patients [19]. Along the same lines, the reported use of vasopressor therapy varies from 62.1% to 100% of cases [17,25]. In the present study, we described a significant association between the need of vasopressor requirement and RRT use.

A study published by Al-Dorzi et al. showed that CRRT use among ICU patients is inevitably linked with a larger decline in hemoglobin levels, requiring red blood cell transfusion [26]. This association has also been identified in our critically ill patients.

Various studied have shown that patients with baseline renal dysfunction (increased serum creatinine values) are at risk of developing AKI during their ICU stay and therefore need RRT. In addition, these patients have an increased risk of mortality [15,27,28]. In our study, patients presenting with previously chronic kidney dysfunction had greater need for RRT and a lower likelihood of survival.

In accordance with previous published studies, LOS is higher in patients receiving RRT [20,21,25], but reports about length of ICU stay in relation to the main organ dysfunction at admission are extremely limited. We noted that multiple trauma and surgical patients, or patients with renal and gastrointestinal dysfunction that needed RRT, had a greater risk of increased ICU stay compared with those without this intervention.

As we emphasized before, there is a large variability among reports about the mortality rate in patients receiving RRT. In patients with AKI receiving continuous-RRT, the mortality rate was 50.6% in a study by Iwagami et al. [29], 58% in a study by Brivet et al. [30], between 54.3–60.7% in patients with CKD or AKI in a report by Allegretti et al. [31], and 66.5% in patients with AKI in a study conducted by Kao et al. [32]. In our study of critically ill patients, 66.8% of patients receiving RRT for AKI or MODS died in ICU, 15% of them dying in the first two days after ICU admission, values almost double compared with patients without this intervention. Interestingly, in patients from the RRT group who presented neurologic dysfunction upon admission, the mortality percentage was lower compared to the non-RRT group.

Worthy of mention is the fact that RRT use significantly influences survival time, and patients requiring RRT during their ICU stay have a risk of fatal outcome 1.62 times higher than those belonging to the Non-RRT group. Another important element is that survival proportion decreases significantly 12 days after ICU admission, both in the RRT and non-RRT groups.

## 5. Limitations

This research has some limitations. First, it is a single-unit, retrospective study using medical records from a database. Therefore, we were unable to identify if certain patients have had RRT only for renal dysfunction or in the context of multisystem organ failure. Second, we could not specify the exact indication nor the exact time for RRT start during the ICU stay (except for patients in whom it was performed in the first 48 h), the RRT modalities, RRT session length or the type of filters used.

## 6. Conclusions

Those between 51 and 80 years of age, of male gender, from general surgery and internal medicine wards, and patients with gastrointestinal, respiratory, cardiac dysfunctions or having a postoperative state at ICU admission have a higher risk of needing CRRT during their ICU stay. High ISS values correlate with the need for RTT in critically ill patients. Patients with multiple traumas, postoperative status, renal, gastrointestinal or cardiac dysfunction requiring RRT during ICU stay have an increased LOS compared with those without RRT. The need of RRT therapy in critically ill patients is associated with increased mortality, both in the short- (48 h) and long-term (during ICU stay), possibly in correlation with the extreme severity of cases resulting in a rapid fatal prognosis despite maximum therapeutic management.

We consider that acknowledging the epidemiological characteristics of the critically ill population in need of RRT and identifying at risk patients (i.e., patients from general surgical or internal medicine wards, those with gastrointestinal or respiratory dysfunctions, and postoperative state at ICU admission), are main steps to improve patient outcomes and to predict trends of medical resources use.

## Figures and Tables

**Table 1:** Patients’ distribution according to age intervals.

Age Interval	RRT Group (*n* = 238)	Non-RRT Group (*n* = 1465)
17–20	4 (1.7%)	17 (1.2%)
21–30	9 (3.8%)	63 (4.3%)
31–40	7 (2.9%)	100 (6.8%)
41–50	25 (10.5%)	185 (12.6%)
51–60	40 (16.8%)	211 (14.4%)
61–70	54 (22.7%)	305 (20.8%)
71–80	61 (25.6%)	323 (22.0%)
81–90	35 (14.7%)	233 (15.9%)
91–100	3 (1.3%)	28 (1.9%)

Results are conferred as numbers (percent). RRT—renal replacement therapy.

**Table 2:** Characteristics of critically ill patients at admission.

Data		RRT Group (*n* = 238)	Non-RRT Group (*n* = 1465)	*p* Value
Age		64.73 ± 16.65 SD	63.40 ± 17.67 SD	*p* = 0.279
Gender	male	149 (62.6%)	881 (60.1%)	*p* = 0.470
	female	89 (37.4%)	584 (39.9%)	
ICU admission from various medical wards	cardiology	14 (5.8%)	88 (6.0%)	*p* = 0.9451
	vascular surgery	4 (1.6%)	6 (0.4%)	*p* = 0.0548
	general surgery	98 (41.1%)	360 (24.5%)	*p* < 0.001
	internal medicine	58 (24.3%)	340 (23.2%)	*p* = 0.7568
	neurosurgery	13 (5.4%)	365 (24.9%)	*p* < 0.001
	neurology	5 (2.1%)	77 (5.2%)	*p* = 0.0514
	orthopedic surgery	21 (8.8%)	125 (8.5%)	*p* = 0.9813
	plastic surgery	10 (4.2%)	31 (2.1%)	*p* = 0.0868
	gastroenterology	14 (5.8%)	73 (4.9%)	*p* = 0.6699
	toxicology	1 (0.4%)	0	-
Primary organ dysfunction at ICU admission *	cardiac dysfunction	27 (11.3%)	153 (10.4%)	*p* = 0.7602
	respiratory dysfunction	45 (18.9%)	253 (17.2%)	*p* = 0.5991
	renal dysfunction	14 (5.8%)	33 (2.2%)	*p* = 0.0031
	gastrointestinal dysfunction	53 (22.2%)	226 (15.4%)	*p* = 0.0108
	neurologic dysfunction	14 (5.8%)	416 (28.3%)	*p* < 0.001
	multiple trauma	24 (10.1%)	158 (10.7%)	*p* = 0.8328
	surgery	36 (15.1%)	140 (9.5%)	*p* = 0.0123
	oncological patients	25 (10.5%)	86 (5.8%)	*p* = 0.0110
Mechanical ventilation	days, median (IQR)	4.5 (9.00)	4 (7.00)	*p* = 0.315
	number of patients (*n* = 1034)	215 (90.3%)	819 (55.9%)	-
Specific ICU scores ^#^ at admission	APACHE II score (points), median (IQR)	21 (7.00)	18 (12.00)	*p* < 0.001
	SOFA score (points), median (IQR)	10 (6.00)	8 (7.00)	*p* < 0.001
	ISS score (points), median (IQR)	43 (20)	26 (16)	*p* = 0.001
ICU stay, days, median (IQR)		8 (11.75)	6 (11.00)	*p* < 0.001
Vasopressor treatment (yes/no) (during ICU stay)		206 (86.5%)	685 (46.7%)	*p* < 0.001
Transfusions (yes/no) (during ICU stay)		151 (63.4%)	397 (27.1%)	*p* < 0.001
RRT sessions, mean ± SD		2.01 ± 0.58	-	-

Notes: All the results are conferred as numbers and percentages, as a median (IQR) or as mean ± SD. RRT—renal replacement therapy, ICU—intensive care unit. * cardiac dysfunction: congestive cardiac failure, acute myocardial infarction, post-cardiac arrest, arrhythmias; respiratory dysfunction: asthma, bronchopneumonia, chronic obstructive pulmonary disease (COPD), respiratory failure; renal dysfunction: AKI, acute-on-chronic kidney disease; gastrointestinal dysfunction: upper or lower gastrointestinal bleedings, perforated ulcer, peritonitis, pancreatitis, intestinal occlusion, cirrhosis, hepatic failure; neurologic dysfunction: neurosurgery, acute cerebrovascular accident; surgery: vascular, orthopedics, plastic and reconstructive, general. ^#^ APACHE II = Acute Physiology and Chronic Health Evaluation, SOFA = Sequential Organ Failure Assessment, ISS = Injury Severity Score.

**Table 3:** The mortality percentage according to primary organ dysfunction.

Primary Organ Dysfunction at ICU Admission *	RRT Group (*n* = 238)	Non-RRT Group (*n* = 1465)	*p* Values for RRT Group
cardiac dysfunction	21 (8.80%)	91 (6.20%)	*p* = 0.1720
respiratory dysfunction	26 (10.90%)	100 (6.80%)	*p* = 0.0356
renal dysfunction	10 (4.20%)	18 (1.20%)	*p* = 0.0022
gastrointestinal dysfunction	32 (13.40%)	82 (5.60%)	*p* < 0.001
neurologic dysfunction	10 (4.20%)	130 (8.90%)	*p* = 0.0211
multiple trauma	16 (6.70%)	24 (1.60%)	*p* < 0.001
surgery	24 (10.10%)	32 (2.18%)	*p* < 0.001
oncologic patients	20 (8.40%)	21 (1.40%)	*p* < 0.001

Notes: All the results are conferred as numbers and percentages. RRT—renal replacement therapy, ICU—intensive care unit. * cardiac dysfunction: congestive cardiac failure, acute myocardial infarction, post-cardiac arrest, arrhythmias; respiratory dysfunction: asthma, bronchopneumonia, chronic obstructive pulmonary disease (COPD), respiratory failure; renal dysfunction: AKI, acute-on-chronic kidney disease; gastrointestinal dysfunction: upper or lower gastrointestinal bleedings, perforated ulcer, peritonitis, pancreatitis, intestinal occlusion, cirrhosis, hepatic failure; neurologic dysfunction: neurosurgery, acute cerebrovascular accident; surgery: vascular, orthopedics, plastic and reconstructive, general.

**Table 4:** The relative instantaneous risks of mortality in our patients.

Variables	b	SE	Wald	*p*	Exp(b)	95% CI of Exp(b)
age	0.01765	0.002751	41.1718	<0.001	1.0178	1.0124 to 1.0233
cardiac dysfunction	0.4950	0.1161	18.1855	<0.001	1.6406	1.3082 to 2.0573
gastrointestinal dysfunction	0.2958	0.1158	6.5274	0.0106	1.3442	1.0725 to 1.6846
male gender	−0.1610	0.08102	3.9511	0.0468	0.8512	0.7268 to 0.9969
neurologic dysfunction	−0.2364	0.1124	4.4237	0.0354	0.7894	0.6341 to 0.9829
multiple trauma	−0.5668	0.1790	10.0262	0.0015	0.5674	0.4002 to 0.8043
renal dysfunction	0.4104	0.2029	4.0891	0.0432	1.5074	1.0147 to 2.2391
RRT	0.4002	0.09518	17.6771	<0.001	1.4921	1.2393 to 1.7964

Notes: The table lists the variables included in the model, their regression coefficient b with standard error (SE), Wald statistic (b/SE)^2^ and associated *p*-value, Exp(b) and the 95% confidence interval for Exp(b); Exp(b): the relative instantaneous risk.

**Table 5:** Survival proportion for various moments (days) during ICU stay.

	RRT Group		Non-RRT Group		Overall	
Survival Time *	Survival Proportion ^#^	Standard Error	Survival Proportion ^#^	Standard Error	Survival Proportion ^#^	Standard Error
1	0.937	0.0158	0.954	0.00550	0.951	0.00522
2	0.848	0.0233	0.901	0.00787	0.894	0.00753
3	0.791	0.0265	0.857	0.00954	0.847	0.00905
4	0.738	0.0288	0.823	0.0107	0.810	0.0101
5	0.702	0.0302	0.796	0.0116	0.781	0.0109
6	0.659	0.0315	0.756	0.0129	0.740	0.0120
7	0.619	0.0326	0.734	0.0135	0.714	0.0126
8	0.583	0.0335	0.712	0.0142	0.689	0.0132
9	0.561	0.0339	0.686	0.0150	0.664	0.0138
10	0.524	0.0345	0.660	0.0158	0.635	0.0145
12	0.478	0.0351	0.622	0.0168	0.595	0.0153
20	0.259	0.0348	0.497	0.0205	0.444	0.0183
25	0.192	0.0330	0.463	0.0215	0.399	0.0191
35	0.170	0.0326	0.354	0.0254	0.313	0.0212
40	0.155	0.0331	0.338	0.0259	0.296	0.0216
50	0.133	0.0350	0.262	0.0286	0.233	0.0236
60	0.0885	0.0346	0.232	0.0300	0.196	0.0249

Notes: All of the results are conferred as numbers. * days, ^#^ percentage. RRT—renal replacement therapy.

**Table 6:** Length of ICU stay according to primary organ dysfunction (days).

Primary Organ Dysfunction at ICU Admission *	RRT Group (*n* = 238)	Non-RRT Group (*n* = 1465)	*p* Values for RRT Group
cardiac dysfunction	7 (14.00)	5 (8.00)	*p* = 0.221
respiratory dysfunction	7 (12.00)	6 (9.00)	*p* = 0.297
renal dysfunction	10 (10.25)	4 (4.50)	*p* = 0.019
gastrointestinal dysfunction	6 (12.50)	4 (6.00)	*p* = 0.006
neurologic dysfunction	7 (10.00)	6 (10.00)	*p* = 0.259
multiple trauma	12.50 (12.75)	8 (12.25)	*p* = 0.041
surgery	7 (11.75)	3 (4.00)	*p* < 0.001
oncologic patients	6 (9.50)	5 (6.00)	*p* = 0.537

Notes: Results are presented as median and (IQR). * cardiac dysfunction: congestive cardiac failure, acute myocardial infarction, post-cardiac arrest, arrhythmias; respiratory dysfunction: asthma, bronchopneumonia, chronic obstructive pulmonary disease (COPD), respiratory failure; renal dysfunction: AKI, acute-on-chronic kidney disease; gastrointestinal dysfunction: upper or lower gastrointestinal bleedings, perforated ulcer, peritonitis, pancreatitis, intestinal occlusion, cirrhosis, hepatic failure; neurologic dysfunction: neurosurgery, acute cerebrovascular accident; surgery: vascular, orthopedics, plastic and reconstructive, general. ICU—intensive care unit; RRT—renal replacement therapy.

## Data Availability

The data used and analyzed in the present study involved private patient information. The database is available, only upon reasonable request, to the corresponding author.
